# Substantia Nigra Echogenicity Signal Correlated with Clinical Features in Patients with Parkinson's Disease in Xinjiang

**DOI:** 10.1155/2023/8867546

**Published:** 2023-06-01

**Authors:** Rurui Wei, Yan Zhang, Peishan Li, Zeheng Xu, La Zhang, Yan He, Qinfen Wu, Youcai Shi, Yang Yuan, Abudula Aisha

**Affiliations:** ^1^The Second Affiliated Hospital of Xinjiang Medical University, Neuroencephalology Clinical Treatment Centre, Xinjiang Key Laboratory of Neurological Disorder Research, Urumqi, Xinjiang, China; ^2^Department of Epidemiology and Health Statistics, College of Public Health, Xinjiang Medical University, Urumqi, Xinjiang, China

## Abstract

**Background:**

Transcranial sonography (TCS) is a noninvasive test that can reveal structural changes in the substantia nigra (SN) in Parkinson's disease (PD). The purpose of this study was to investigate the relationship between SN signatures and clinical features in PD patients in a multiethnic region of China.

**Methods:**

A total of 147 patients with PD were included in the study, and all of whom had underwent a TCS examination. Clinical information was collected from PD patients, and motor and nonmotor symptoms were assessed using assessment scales.

**Results:**

There were differences in the substantia nigra hyperechogenicity (SNH) area between age of onset, visual hallucinations (VH), and UPDRS3.0 II scores (*P* < 0.05), patients with late onset PD had a greater SNH area than early onset (0.326 ± 0.352 vs. 0.171 ± 0.194), and PD patients presenting with VH had a greater SNH area than those without hallucinations (0.508 ± 0.670 vs. 0.278 ± 0.659), and further multifactorial analysis showed that a high SNH area was an independent risk factor for development of VH. The area under the ROC curve for predicting VH from the SNH area in PD patients was 0.609 (95% CI: 0.444–0.774). There was a positive correlation between the SNH area and UPDRS3.0-II scores, but further multifactorial analysis showed that SNH was not an independent predictor of the UPDRS3.0-II score.

**Conclusion:**

A high SNH area is an independent risk factor for development of VH, there is a positive correlation between the SNH area and UPDRS3.0 II score, and TCS has guiding significance in predicting clinical VH symptoms and activities of daily living in PD patients.

## 1. Introduction

Parkinson's disease (PD) is a common degenerative movement disorder of the nervous system. The pathological changes are the progressive degeneration of nigrostriatal dopaminergic neurons in the substantia nigra and formation of Lewy vesicles [1]. It is characterized by motor symptoms and nonmotor symptoms as the main clinical manifestation. Bradykinesia, resting tremor, muscle rigidity, abnormal posture, and gait are the main manifestations of motor symptoms, and motor complications are often accompanied by middle and late stages of the disease. Its diagnosis is based on the presence of parkinsonian motor features, namely, bradykinesia, stiffening, and resting tremor, and the early misdiagnosis rate is high.

Transcranial sonography (TCS) is a noninvasive, convenient, and rapid examination that utilizes weak areas of the skull as transillumination windows and allows ultrasound exploration of the intracranial vessels and structures in the substantia nigra of the midbrain using the Doppler effect of ultrasound. Since Becker [2] first described the substantia nigra hyperechogenicity (SNH) in PD patients in 1995, a large number of studies have reported that TCS can be used as one of the imaging markers of PD, providing auxiliary evidence for the early diagnosis of PD and assisting in the identification of other movement disorder diseases. Several previous studies [3–7] have shown the differences in sensitivity, specificity, and clinical characteristics of TCS in Asian and European patients with PD, and Xinjiang is located in the hinterland of Asia and Europe, which is a multiethnic region with good ethnic diversity. This study intends to further investigate the correlation between SNH and the clinical characteristics of PD patients through clinical studies and provide reference information for the diagnosis and treatment of PD patients.

## 2. Materials and Methods

### 2.1. Research Subjects

This is a cross-sectional study with subjects from the Second Affiliated Hospital of Xinjiang Medical University, which is a clinical medical research center for neurological disorders in Xinjiang, with patients with neurological disorders from various states in Xinjiang and high ethnic diversity. Patients with PD who attended the Neurology Treatment Centre of the Second Affiliated Hospital of Xinjiang Medical University from January 2021 to October 2022 were selected. All patients were diagnosed by two experienced neurologists according to the diagnostic criteria for primary Parkinson's disease [8], and possible dementia with Lewy bodies (DLB) was excluded according to the 2017 DLB diagnostic criteria (DLBC-4) [9]. Possible secondary Parkinson's disease due to cone signs, gaze palsy, cerebellar ataxia, history of stroke, neuroleptic intake, or other possible causes was also ruled out. Two doctors were double blinded during the consultation and excluded the patient if a consistent diagnosis could not be made. All patients had MR/CT brain scans to aid in the diagnosis, and no patients underwent SPECT or PET imaging to refine the detection of dopamine transporters or F-dopa. A total of 161 patients with PD were included in the study. After completion of TCS in all patients, 14 patients were excluded due to the inability to assess the nigrostriatal echogenic signal in both temporal windows conditions, and finally, 147 patients were included in this study. The study was approved by the Ethics Committee of the Second Affiliated Hospital of Xinjiang Medical University, and all patients signed informed consent.

### 2.2. Transcranial Sonography

The patient's TCS was performed by an experienced ultrasonographer who had no knowledge of the patient's clinical profile. The examination equipment was a Resona 7T color Doppler ultrasound diagnostic instrument (Shenzhen, China), set at a scanning depth of 16 cm and an acquisition range of 45 dB. The examining physician performed the procedure at the typical temporal bone window above the zygomatic arch in the preauricular region and adjusted the probe angle to find the best imaging effect in the butterfly-shaped midbrain and finally measured the nigrostriatal high signal area automatically with a cursor. The study used both semiquantitative grading criteria [2] and quantitative criteria (area) [10] of the substantia nigra signal to assess the substantia nigra properties. The semiquantitative grading criteria were grade I: the substantia nigra showed uniformly distributed hypoechogenicity; grade II: scattered dotted and fine-linear slightly strong echoes were seen in the substantia nigra; grade III: the substantia nigra echoes were patchily enhanced and lower than the foot interrogation pool echoes; grade IV: the substantia nigra echoes were patchily enhanced and equal to the foot interrogation pool echoes; grade V: the substantia nigra echoes were patchily enhanced and higher than the foot interrogation pool echoes. SNH above grade III and/or an area ≥0.2 cm^2^ was considered positive nigrostriatal (SN+).

### 2.3. Clinical Assessment

Relevant clinical characteristics of the study population were collected: race, age, sex, education level, and duration of disease. The Uniform Parkinson's Disease Rating Scale (UPDRS) published in 1987 [11] and the Hoehn and Yahr Staging (H–Y staging) were used to assess the severity of Parkinson's disease in patients after 24 hours (or 72 hours for controlled-release anti-PD drugs) of discontinuation of treatment with anti-Parkinson's disease drugs. The Non-Motor Symptom Scale (NMSS) was used to assess nonmotor features such as bladder status, constipation, and pain in PD patients, the Montreal Cognitive Assessment Scale (MoCA) was used to assess cognitive function in PD patients, and the Hamilton Depression Scale (HAMD) and the Hamilton Anxiety Scale (HAMA) were used to assess depression and anxiety status of PD patients, respectively. The Montreal Cognitive Assessment Scale (MoCA) ≥26 is normal, ≥18 is mild cognitive impairment, ≥17 is moderate, and <10 is severe. The Hamilton Depression Scale (HAMD) <7 is normal, ≥7 is possible depression, ≥17 is definitely depression, and >24 is severe depression. The Hamilton Anxiety Scale (HAMA) ≥29 may be severe anxiety, ≥21 is definitely obvious anxiety, ≥14 points is definitely anxiety, ≥7 points is possible anxiety, and <7 is normal. The study further classified PD patients into early-onset PD (≤50 years old) and late-onset PD (>50 years old) according to Chinese guidelines [12].

### 2.4. Statistical Analysis

EXCEL software was used for data collection and organization, and SPSS 21.0 statistical software was used for statistical data analysis. Continuous variables were described by means and standard deviations $\left(\overset{®}{x}\pm s\right)$, and categorical variables were described by frequencies and percentages n(n%). Comparisons of the SNH area between dichotomous variables (gender, voiding disorder, etc.) were performed using the *t*-test or Mann–Whitney *U* test. Correlation analysis between quantitative variables (UPDRS 3.0 I score, improvement rate, etc.) and the nigrostriatal high-signal area was performed using the Pearson correlation test. Logistic regression analysis of univariate and multivariate factors was used for the analysis of factors influencing whether visual hallucinations (VH) occurred in PD patients, and linear regression analysis was used for the analysis of factors influencing the UPDRS3.0 II score. The significant level was set at *α* = 0.05.

## 3. Results

### 3.1. Description of Clinical Characteristics of 147 Patients with PD

Of the 147 PD patients included in the study, 85 (57.8%) were male, 97 (66.0%) were Han nationality, 25 (17.0%) were Uyghurs, 14 (9.5%) were Kazakhs, 9 (6.1%) were Hui nationality, 1 (0.68%) was Kyrgyz, and 1 (0.68%) was Mongolian; 125 (85%) had an age of onset >50 years. The mean duration of disease in PD patients in the study was 5.267 ± 3.913 years. The SNH area of PD patients was 0.454 ± 0.319 cm^2^, and 89 (60.5%) had SN + PD. Other relevant basic clinical characteristics are shown in Table 1.

### 3.2. Analysis of Factors Influencing the Area of SNH in PD Patients

**Figure 1:**
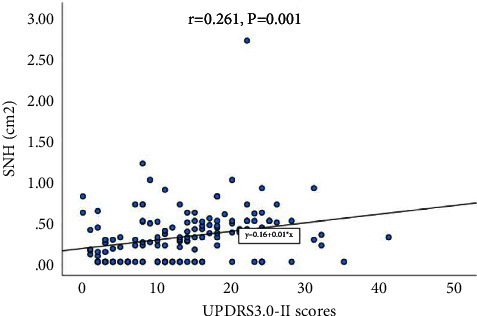
Scatterplot of UPDRS 3.0 II scores versus area in PD.

There were no differences in the SNH area between gender, ethnicity, urinary disturbance, constipation, pain, sleep disturbance, hyposmia, hyperhidrosis, HAMD, HAMA, and cognitive impairment (*P* > 0.05), differences in the SNH area between the age of onset, VH, and UPDRS3.0 II scores (*P* < 0.05), the SNH area was greater in patients with late-onset PD than early onset PD (0.326 ± 0.352 vs. 0.171 ± 0.194), and the SNH area was greater in PD patients presenting with VH than without hallucinations (0.508 ± 0.670 vs. 0.278 ± 0.659), see Table 2. Analysis of the correlation between the SNH area and clinical characteristics in PD patients showed that there was a positive correlation between the SNH area and the UPDRS3.0-II score (*r* = 0.261; *P* value = 0.001), as shown in Table 3 and Figure 1.

### 3.3. Analysis of Factors Influencing VH in PD Patients

**Figure 2:**
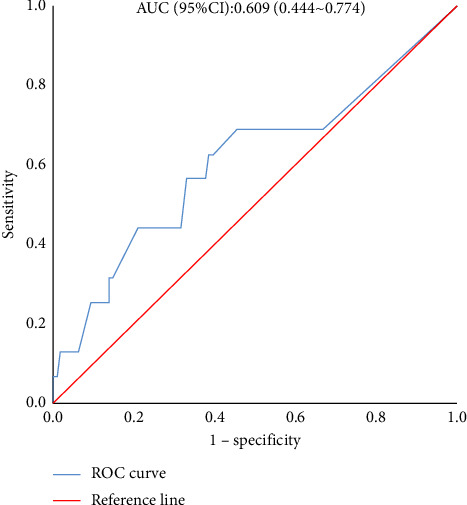
ROC curve of the SNH area predicting VH in PD patients.

To clarify whether the SNH area was an independent risk factor for VH, the study analyzed its influencing factors with VH as the outcome variable, and the results of the single-factor analysis showed that SNH area, UPDRS3.0 I, and H-Y staging were correlated with VH (*P* < 0.05); further multifactor analysis showed that, controlling for constant UPDRS3.0-I and H-Y staging, the SNH area still correlated with VH (OR = 59.661; 95% CI = 2.424∼68.675; *P* = 0.012), and a high SNH area was an independent risk factor for the development of VH, see Table 4. The ROC curve for predicting VH from the SNH area in PD patients yielded an area under the curve (AUC) of 0.609 (95 CI%: 0.444∼0.774), with a cutoff value of 0.345 cm^2^, see Figure 2.

### 3.4. Analysis of Factors Influencing UPDRS 3.0 II Scores in PD Patients

The study analyzed the factors influencing the UPDRS3.0 II score as an outcome variable, and the results of the univariate analysis showed that SNH area, disease duration, voiding disorder, constipation, cognitive impairment, UPDRS3.0 I, UPDRS3.0 III, UPDRS3.0 IV, H-Y staging, and MoCA were correlated with the UPDRS3.0 II score (*P* < 0.05), and further multifactorial analysis showed that UPDRS3.0 III and H-Y staging still had positive correlations with UPDRS 3.0 II scores (*β* > 0; *P* < 0.05), as shown in Table 5.

## 4. Discussion

Of the 161 PD patients collected in the study, 14 (8.6%) were excluded due to poor sound transmission through the temporal bone window, and no nigrostriatal-related information was obtained, which is consistent with the 6.9%–15.5% [5] rate reported in the literature for Caucasians and inconsistent with a reported rate of 20.5%–30.2% in Asians [4]. The results of the study [4] showed that the proportion of women in the group with poor temporal bone ultrasound penetration was significantly higher than that in the group with good temporal bone ultrasound penetration (74.71% vs. 30.85%, *P* < 0.001). It has also been shown [13] that frontal lobe hypertrophy develops with age, particularly in females. In contrast, the relatively high proportion of males in this study, with a male-to-female ratio of (57.8% vs. 42.2%), may have contributed to the lower proportion of poor ultrasound penetration of the temporal bone in this study. In addition, the ethnic diversity of this study may have contributed to some degree of disparity. The proportion of 147 PD patients with SNH above grade III and/or an area of 0.2 cm^2^ or more was 60.5%, which is much less sensitive than earlier reports in the literature [6] and not inconsistent with reports of similar sensitivity in Asians and Europeans [7] but similar to the reported 61.69% in Chinese [4]. This may be related to a variety of factors such as the selection of the optimal section during TCS examination related to operator experience, different ultrasound equipment accuracy, subjectivity in the determination of nigrostriatal echo intensity, human error in the calculation of the SN + area, and the diverse pathogenesis of PD patients.

This study investigated the correlation between clinical characteristics and the SNH area in 147 PD patients, and it was found that the SNH area was not related to gender and ethnicity but to age of onset, VH, and UPDRS II scores. The study divided PD patients into two groups, early-onset and late-onset, and the SNH area of late-onset PD patients was significantly higher than that of early-onset PD patients (*P* < 0.05). The mechanism may be the accumulation of various metallic elements in the midbrain substantia nigra with age, with iron ions, manganese ions, and calcium ions [14], which are thought to be responsible for SNH. The age of onset and the echogenicity of the substantia nigra are still highly controversial. It has been reported in the literature [15] that younger PD patients have larger hyperechoic areas than older PD patients and that SNH is positively correlated with age in healthy individuals. The Berg [16] study, on the other hand, suggests that the odds of SNH gradually increase with age. It has also been suggested [17] that the area of SNH increases with the severity of the disease after 5 years of patient onset. The mean duration of disease in PD patients in the study was 5.267 ± 3.913 years, and no correlation between the duration of disease and SN echo was found, probably due to the relative difficulty of early diagnosis of PD and some error in the definition of the duration of disease, and further follow-up studies are still needed.

16 of 147 patients with PD had VH, with a prevalence of 10.9%, which is consistent with 8.8%–44% [18, 19] reported in the literature. The mechanism of VH is not fully understood, and previous studies [3, 20, 21] have shown that VH is associated with disease duration, cognitive dysfunction, sleep quality, anticholinergic drugs, and severity (H-Y staging). The present study confirmed that VH was associated with SNH, UPDRS I scores, and H-Y staging. The study showed that the area of SNH in patients with VH was significantly larger than that without VH, and further multifactorial analysis revealed that a high SNH area was an independent risk factor for VH, which is consistent with the results of related studies. The main mechanisms associated with the occurrence of SNH in PD patients include iron deposition [1] and microglia activation [22]. There is no direct evidence that either iron accumulation in the substantia nigra or microglia activation is associated with PD with VH, so the mechanism of SNH area associated with VH needs further study. At the same time, the study used the ROC curve to determine the value of the SNH area to predict VH, and the AUC value was 0.609, suggesting that the value of the SNH area alone to predict VH is low; the SNH area still needs to be combined with other relevant factors for VH prediction. In addition, it was found that after dividing all PD patients into VH and no VH groups, all PD patients in the VH group had late onset of VH, but it was not related to the duration of the disease, indicating that the patients' age of onset was related to the occurrence of VH, which is consistent with related studies [21, 23]. This may be related to rapid sensory loss and age-related drug side effects [24–26]. This study found a correlation between VH and the UPDRS I score, which may be related to the fact that the UPDRS I score is mainly used to determine the degree of mental activity and behavioral and emotional disturbances in patients with PD.

Several previous studies [4, 27] have confirmed the correlation between SNH and the UPDRS II score, which has a clear correlation with disease duration and severity (H-Y staging), but SNH did not show a clear correlation with disease duration and severity (H-Y staging) in the present study. The study found that the UPDRS III score and severity (H-Y stage) were independent risk factors for the UPDRS II score by further multifactorial analysis, while the area was not an independent risk factor, and the contradictory relationship between them was not well understood, probably because different PD subtypes lead to different mechanisms of SNH; further exploratory studies are needed.

## 5. Limitations

There are still some methodological limitations to this study. First, due to the number of factors contributing to hallucinations, although the study population was assessed 24 hours after stopping the drug, the effects of the drug could still not be completely excluded. Second, given the small sample size and ethnic diversity ratio in this study, statistical power remains limited, and a larger multiethnic sample size is needed to validate the current work.

## 6. Conclusion

TCS, as a noninvasive test, has a certain value in the diagnosis of PD patients. The study found that a high SNH area was an independent risk factor for the development of VH by statistical analysis of the SNH area and clinical characteristics of PD, and there was a positive correlation between the SNH area and the UPDRS3.0 II score. TCS has some guiding significance in predicting clinical VH symptoms and activities of daily living in PD patients.

## Figures and Tables

**Table 1:** Description of clinical characteristics of 147 PD patients.

Clinical features	Statistical description *n*(*n*%)/$\overset{®}{x}$ ± *s*
Gender: male	85 (57.8)
Ethnicity: Han nationality	97 (66.0)
Age of onset: >50 years	125 (85)
SNH (cm^2^)	0.454 ± 0.319
SN+	89 (60.5)
Course of disease (years)	5.267 ± 3.913
UPDRS3.0 I	3.151 ± 2.888
UPDRS3.0 II	13.231 ± 8.384
UPDRS3.0 III	27.457 ± 13.129
UPDRS3.0 IV	1.583 ± 2.475
H-Y staging	2.605 ± 0.977
VH	16 (10.9)
Urination disorders	64 (43.8)
Constipation	97 (66.0)
Pain	25 (17.2)
Sleep disorders	95 (65.5)
Hyposmia	29 (20.0)
Hyperhidrosis	21 (14.5)
Cognitive impairment	44 (33.8)
HAMD	54 (41.5)
HAMA	30 (23.1)
MoCA	20.65 ± 5.887

**Table 2:** Analysis of factors influencing the SNH area in PD patients.

Clinical features	SNH area (cm^2^)		*t* value	*P* value
*Gender*				
Female	0.276 ± 0.393		−0.833	0.406
Male	0.323 ± 0.291			

*Ethnicity*				
Han nationality	0.327 ± 0.362		1.106	0.270
Minority nationality	0.261 ± 0.280			

*Age of onset (years)*				
>50	0.326 ± 0.352		2.003	0.047
≤50	0.171 ± 0.194			

*Urination disorders*				
No	0.321 ± 0.391		0.720	0.473
Yes	0.281 ± 0.256			

*Constipation*				
No	0.308 ± 0.292		0.099	0.921
Yes	0.302 ± 0.361			

*Pain*				
No	0.310 ± 0.342		0.593	0.554
Yes	0.266 ± 0.328			

*Sleep disorders*				
No	0.291 ± 0.280		−0.272	0.786
Yes	0.307 ± 0.367			

*Hyposmia*				
No	0.307 ± 0.354		0.401	0.689
Yes	0.279 ± 0.274			

*Hyperhidrosis*				
No	0.303 ± 0.345		0.100	0.921
Yes	0.295 ± 0.305			

*VH*				
No	0.278 ± 0.659		−2.632	0.009
Yes	0.508 ± 0.670			

*HAMD*				
No	0.355 ± 0.391		1.613	0.109
Yes	0.255 ± 0.281			

*HAMA*				
No	0.344 ± 0.369		1.818	0.071
Yes	0.212 ± 0.265			

*Cognitive impairment*				
No	0.288 ± 0.285		−1.089	0.278
Yes	0.359 ± 0.453			

**Table 3:** Correlation analysis of the SNH area and clinical characteristics of PD patients.

Clinical features	Correlation coefficient (*r*)	*P* value
Course of disease	−0.129	0.118
UPDRS3.0 I	0.020	0.852
UPDRS3.0 II	0.261	0.001
UPDRS3.0 III	−0.182	0.083
UPDRS3.0 IV	−0.053	0.633
H-Y staging	0.020	0.815
MoCA	0.014	0.890

**Table 4:** Analysis of factors influencing VH in PD patients.

Clinical features	VH		Single-factor analysis		Multifactor analysis	
	No	Yes	OR (95% CI)	*P* value	OR (95% CI)	*P* value
SNH (cm^2^)	0.276 ± 0.263	0.521 ± 0.670	4.380 (1.111, 17.266)	0.035	59.661 (2.424, 68.675)	0.012
UPDRS 3.0 I	2.924 ± 2.551	5.714 ± 5.024	1.271 (1.027, 1.574)	0.027	1.189 (0.906, 1.560)	0.213
H-Y staging	2.557 ± 0.978	3.000 ± 0.906	1.842 (1.061, 3.199)	0.030	1.693 (0.506, 5.669)	0.393

**Table 5:** Analysis of factors influencing UPDRS 3.0 II scores of PD patients.

Clinical features	Single-factor analysis		Multifactor analysis	
	*β* value	*P* value	*β* value	*P* value
SNH (cm^2^)	0.261 (2.561, 10.440)	0.001	0.048 (−2.729, 5.647)	0.487
Course of disease	0.292 (0.288, 0.961)	<0.001	0.160 (−0.044, 0.915)	0.074
Urination disorder (reference: no)	0.241 (1.363, 6.766)	0.003	0.051 (−1.916, 3.770)	0.516
Constipation (reference: no)	0.179 (0.299, 6.054)	0.031	0.048 (−1.974, 3.777)	0.532
Cognitive impairment (reference: no)	0.415 (4.604, 10.323)	<0.001	0.048 (−4.257, 6.089)	0.724
UPDRS3.0 I	0.567 (1.170, 2.245)	<0.001	0.085 (−0.310, 0.933)	0.319
UPDRS3.0 III	0.743 (0.404, 0.592)	<0.001	0.433 (0.154, 0.409)	<0.001
UPDRS3.0 IV	0.426 (0.821, 2.258)	<0.001	0.014 (−0.574, 0.677)	0.869
H-Y staging	0.460 (2.694, 5.317)	<0.001	0.223 (0.365, 4.178)	0.020
MoCA	−0.454 (−0.906, −0.400)	<0.001	0.028 (−0.350, 0.444)	0.813

## Data Availability

The data that support the findings of this study are available on request from the corresponding author.

## Abbreviations

TCS: Transcranial sonography
SN: Substantia nigra
PD: Parkinson's disease
SNH: Substantia nigra hyperechogenicity
VH: Visual hallucinations
AUC: Area under the curve.
